# The predictive value and the correlation of peripheral absolute monocyte count, tumor-associated macrophage and microvessel density in patients with colon cancer

**DOI:** 10.1097/MD.0000000000010759

**Published:** 2018-05-25

**Authors:** Zhigui Li, Zhaofen Xu, Yuqian Huang, Rui Zhao, Yaping Cui, Yong Zhou, Xiaoting Wu

**Affiliations:** a Department of Gastrointestinal Surgery, West China Hospital, Sichuan University, Chengdu; b Department of Pathology, The Second People's Hospital of Neijiang City, Sichuan Province, China.

**Keywords:** colon cancer, colorectal cancer, microvessel density, monocyte, tumor-associated macrophage

## Abstract

The tumor microenvironment plays a pivotal role in cancer progression. The purpose of the present study was designed to evaluate the predictive value of peripheral absolute monocyte count, tumor-associated macrophage, microvessel density, and to clarify the correlation between them in patients with colon cancer.

A series of 216 patients with colon cancer were enrolled in this study. The peripheral absolute monocyte count was obtained from preoperative routine blood test. Tumor-associated macrophage and microvessel density were assessed on tissue microarray by immunohistochemistry.

The one, three, five-year overall survival rate for the low absolute monocyte count group was 98.4%, 91.1%, 87.1%, respectively; and for the high absolute monocyte count group was 94.6%, 83.7%, 77.2%, respectively (*P = *.046). The one, three, five-year progression-free survival rate for the low absolute monocyte count group was 94.4%, 87.1%, 85.5%, respectively; and for the high absolute monocyte count group was 90.2%, 75.0%, 73.9%, respectively (*P = *.024). Univariate and multivariate analysis showed that there was a strong association between peripheral monocyte count and clinical outcome. The correlation between peripheral absolute monocyte count, tumor-associated macrophage, and microvessel density were not observed.

The peripheral absolute monocyte count was an independent prognostic factor for overall survival and progression-free survival in colon cancer. The high absolute monocyte count was significantly associated with poor outcome.

## 1 Introduction

Colon cancer represented the major cause of cancer-related mortality worldwide,^[1]^ with the growing incidences in many countries.^[2]^ The 5-year and 10-year relative survival rates for patients with colon cancer were 65% and 58%, respectively.^[3]^ Until now, the most important prognostic factor for colon cancer was clinical stage at the time of diagnosis.^[3,4]^ However, this predictive factor was insufficient to predict the outcome accurately. At present, an active area of study focused on how to distinguish the subset of patients who were at high risk of recurrence and metastasis.

It was well known that systemic inflammation plays a pivotal role in the carcinogenesis and cancer progression. Because inflammatory response correlated with alterations of peripheral blood leukocytes, the correlation between peripheral monocyte count and cancer has garnered increased attention. The peripheral absolute monocyte count (AMC) has been demonstrated to correlate with the outcome in various malignancies.^[5–7]^ Tumor-associated macrophages (TAMs) derived from the peripheral monocytes were divided into 2 subgroups: the M1 macrophages responsible for inflammation, antitumor and immune response; the M2 macrophages involving in cancer progression^[8]^ and angiogenesis.^[9,10]^ Microvessel density (MVD) counting the endothelial vessels and measuring their density, was considered to be one of the representative and useful marker of angiogenesis. Several studies demonstrated that the MVD can predict prognosis in various malignancies, such as lung cancer,^[11]^ colorectal cancer^[12]^ and breast cancer.^[13]^ These studies presented the hypothesis that the peripheral monocyte count reflects the density of TAMs which was associated with MVD in the tumor microenvironment.^[4,14–16]^ Nevertheless, these evidences were insufficient to draw the consistent conclusion. The role and the correlation between AMC, TAMs, and MVD have not yet been elucidated in colon cancer.

Therefore, the purpose of the present study was designed to evaluate the predictive value of AMC, TAMs, and MVD and to clarify the correlation between them in patients with colon cancer.

## 2 Materials and methods

### 2.1 Patients

In our study, a total of 216 patients with colon cancer were recruited between February 2012 and March 2013 from the Department of Gastrointestinal Surgery, West China Hospital. The study was approved by the Ethics Committee of West China Hospital of Sichuan University. If patients had inflammatory bowel disease, or received preoperative therapy, or underwent emergency surgery for perforation/obstruction, they were excluded from this study. All patients were followed-up every 3 months in the first postoperative year, every 6 months in the subsequent year until October 2017 or death. The follow-up period was defined as the duration of time from the date of surgery to the date of the last known contact or the date of death. Progression-free survival (PFS) was calculated from the date of surgery to the date of recurrence or metastasis. Overall survival (OS) was calculated from the date of surgery to the date of death by any causes. The last follow-up date was the end of October 2017.

### 2.2 Blood sample analysis

Preoperative routine blood test and differential leukocyte count were obtained within 2 weeks before surgery. AMC was calculated by total leukocyte count multiply the percentage of monocytes. The low AMC group was defined as AMC < 0.35×10^9^/L based on the average value.

### 2.3 Tissue microarray and Immunohistochemistry

All tumor samples obtained from West China Hospital Biobank of Sichuan University were made into the donor paraffin blocks. Paraffin sections were initially evaluated by hematoxylin and eosin staining and the representative areas of tumor tissue were marked in the donor paraffin blocks. Using a specialized manual tissue arrayer, 1.5 mm diameter cylinders were sampled from these marked regions and then placed into an empty paraffin block. CD68 has been used as a specific marker to identify TAMs, CD34 to identify MVD. Immunohistochemistry of tissue microarray was performed as follows: 4 μm thick paraffin sections were deparaffinized in xylene, rehydrated, and washed in phosphate buffered saline (PBS) for 10 minutes, thrice. After antigen retrieval was implemented in an autoclave for 3 minutes and 3% hydrogen peroxide solution was employed to block endogenous peroxidase activity, sections were incubated with the primary antibody at 4° centigrade overnight. Subsequently, the sections were thoroughly rinsed with PBS, incubated with secondary antibodies. The immunohistochemical reaction was visualized with 3,3′-diaminobenzidine tetrahydrochloride and counterstained with hematoxylin.

### 2.4 Morphometrical assay

An image analysis system (Olympus upright microscope-BX53, Japan) was utilized. In tumor sections, 3 nonoverlapping, most immunostained fields (hot spots) were selected at low magnification (×100). Individual TAMs were counted at ×400 magnification, MVD was counted at ×200 magnification. TAMs and MVD were evaluated by 2 independent pathologists.

### 2.5 Statistical analysis

All statistical analyses were performed using SPSS 19.0 software (SPSS, Inc., Chicago, IL), and 2-sided *P* value of < .05 was considered statistically significant. We used Spearman's analysis to calculate the correlations of AMC, TAMs, and MVD, Kaplan–Meier method to analyze the OS and PFS. Covariates with a *P* value ≤.10 in the univariable analysis were included in the multivariable analysis. Multivariate Cox proportional hazards regression analysis was employed to evaluate the prognostic factors.

## 3 Results

**Figure 1:**
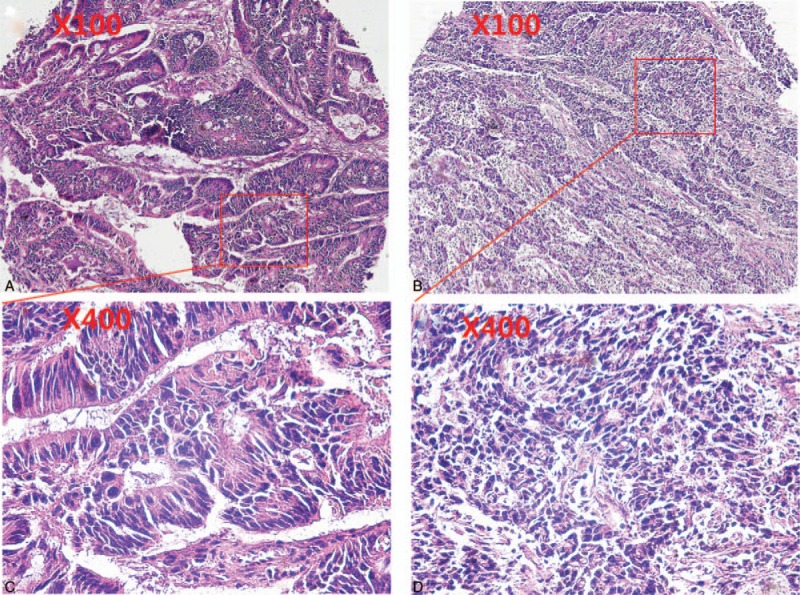
Hematoxylin and eosin staining for tumor tissue. A. middle differentiated adenocarcinoma (×100). B: poorly differentiated adenocarcinoma (×100). C. middle differentiated adenocarcinoma (×400), D: poorly differentiated adenocarcinoma (×400).

**Figure 2:**
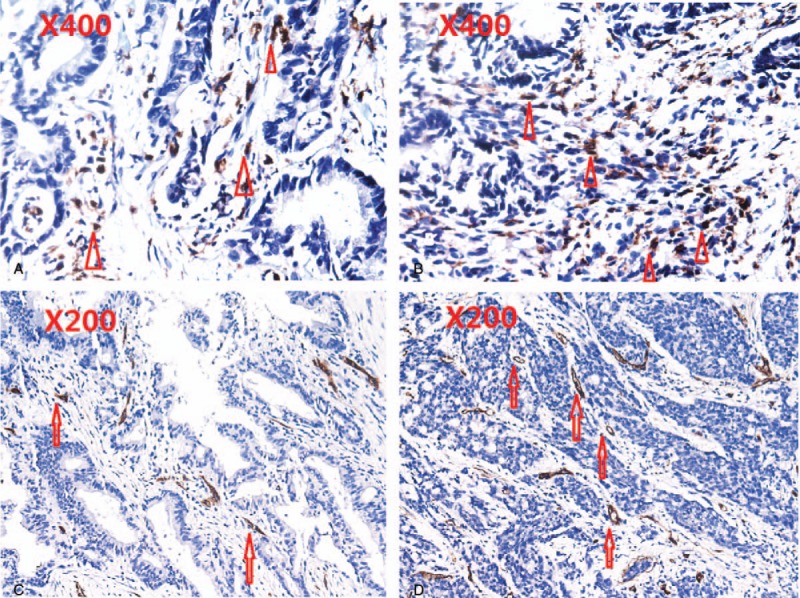
CD68 staining for TAMs and CD34 staining for intratumoral MVD in colon cancer sections. The apex of triangle pointed to TAMs. The arrowhead pointed to MVD. (A) Low TAMs (×400); (B) High TAMs (×400); (C) low MVD (×200); (D) high MVD (×200).

**Figure 3:**
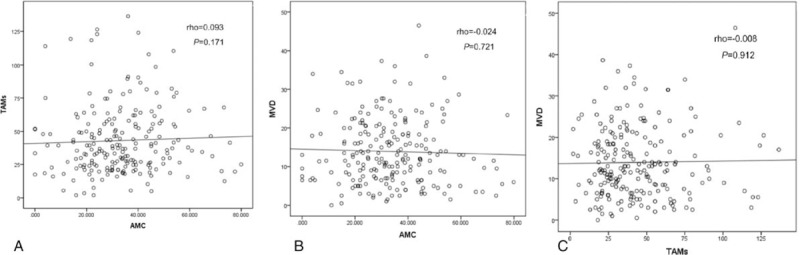
The correlation between AMC, TAMs, and MVD. MVD = microvessel density, TAMs = tumor-associated macrophages.

In the present study, a total of 216 patients with stage I to III colon cancer were included. Details about patient's information were shown in Table 1. Of these, there were 125 males and 91 females. The median age of the patients at the initial surgery was 64 years old (range: 22–89 years old). Around 127 patients had primary tumors located in the right colon, and 89 had primary tumors located in the left colon. There were 68 patients with poorly differentiated adenocarcinoma and 148 patients with middle or well differentiated adenocarcinoma (Fig. 1A–D). The distributions of clinical stages were as follows: stage I, 18 patients; stage II, 131 patients; stage III, 67 patients. The median follow-up from surgery was 60 (5–69) months. During the follow-up period, 37 out of 216 patients were dead at the last follow-up time. At diagnosis, the median AMC (quartile range) was 0.32 (0.22–0.44) ×10^9^/L. In tumor tissue, the median (quartile range) of TAMs, MVD was 37.0 (24.5–55.5), 12.0 (7.0–19.0), respectively (Fig. 2A–D). No significant correlations between AMC and TAMs (*r* = 0.093, *P = *.171), AMC and MVD (*r* = −0.024, *P = *.721), TAMs and MVD (*r* = −0.008, *P = *.912) were found (Fig. 3A–C).

**Table 1:**
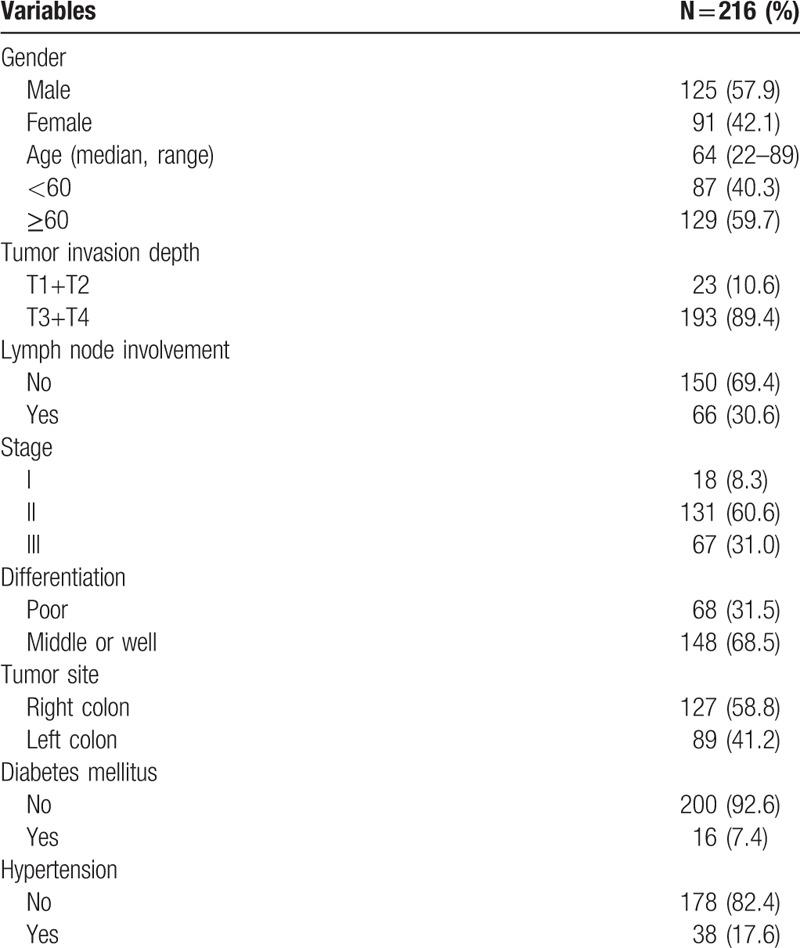
Patient demographics of colon cancer.

**Figure 4:**
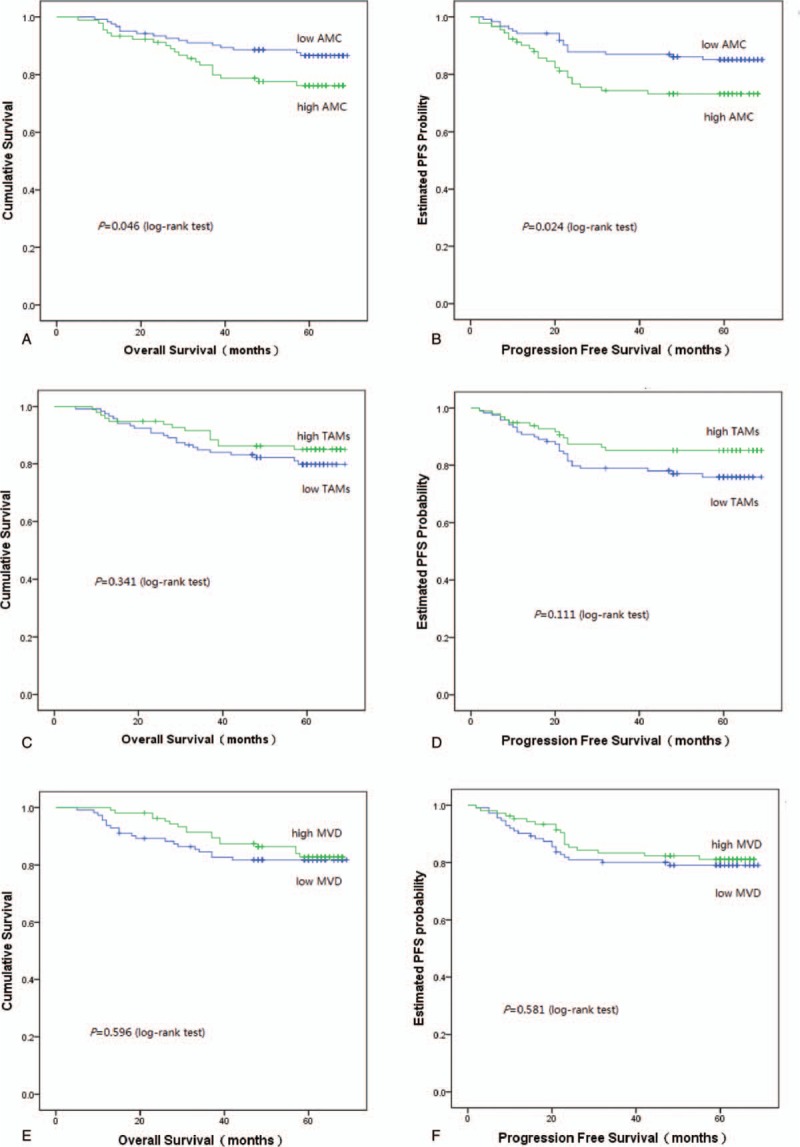
Kaplan–Meier survival curves for OS and PFS according to AMC, TAMs and MVD. (A) The OS rate was significantly better in the low AMC group than in the high AMC group. (B) The PFS rate was significantly better in the low AMC group than in the high AMC group. (C) There was no significant difference in OS rates according to TAMs. (D) There was no significant difference in PFS rates according to TAMs. (E) There was no significant difference in OS rates according to MVD. (F) There was no significant difference in PFS rates according to MVD. AMC = absolute monocyte count, MVD = microvessel density, OS = overall survival, PFS = progression-free survival, TAMs = tumor-associated macrophages.

When all patients were divided into 2 groups by AMC, the 1, 3, 5-year OS rate for the low AMC group was 98.4%, 91.1%, 87.1%, respectively; and for the high AMC group was 94.6%, 83.7%, 77.2%, respectively (log-rank test, *P = *.046). During the follow-up period, 16 (12.9%) were dead in the low AMC group; 21 (22.8%) were dead in the high AMC group (Fig. 4A). The 1, 3, 5-year PFS rate for the low AMC group was 94.4%, 87.1%, 85.5%, respectively; and for the high AMC group was 90.2%, 75.0%, 73.9%, respectively (log-rank test, *P = *.024). During the follow-up period, 18 (14.5%) had metastatic disease in the low AMC group; 24 (26.1%) had metastatic disease in the high AMC group (Fig. 4B).

The low TAMs group was defined as TAMs < 40 based on the average value. When all patients were divided into 2 groups by TAMs, there were no differences in the OS rates (log-rank test, *P = *.341) and the PFS rates (log-rank test, *P = *.111) (Fig. 4C and D). Similarly, when the low MVD was defined as MVD < 13 based on the average value, all patients were divided into 2 groups by MVD, there were no differences in the OS rates (*P = *.596) and PFS rates (*P = *.581) (Fig. 4E and F).

In the univariate analysis, lymph node involvement, clinical stage, differentiation grade, diabetes mellitus, AMC significantly correlated with the OS and PFS. Because of clinical stage based on tumor invasion depth, lymph node involvement and metastasis, only clinical stage entered the multivariate analysis. The multivariate analysis showed that there was a significant association between clinical stage, differentiation grade, AMC, with OS (Table 2) and PFS (Table 3). The low AMC was significantly associated with better outcome.

**Table 2:**
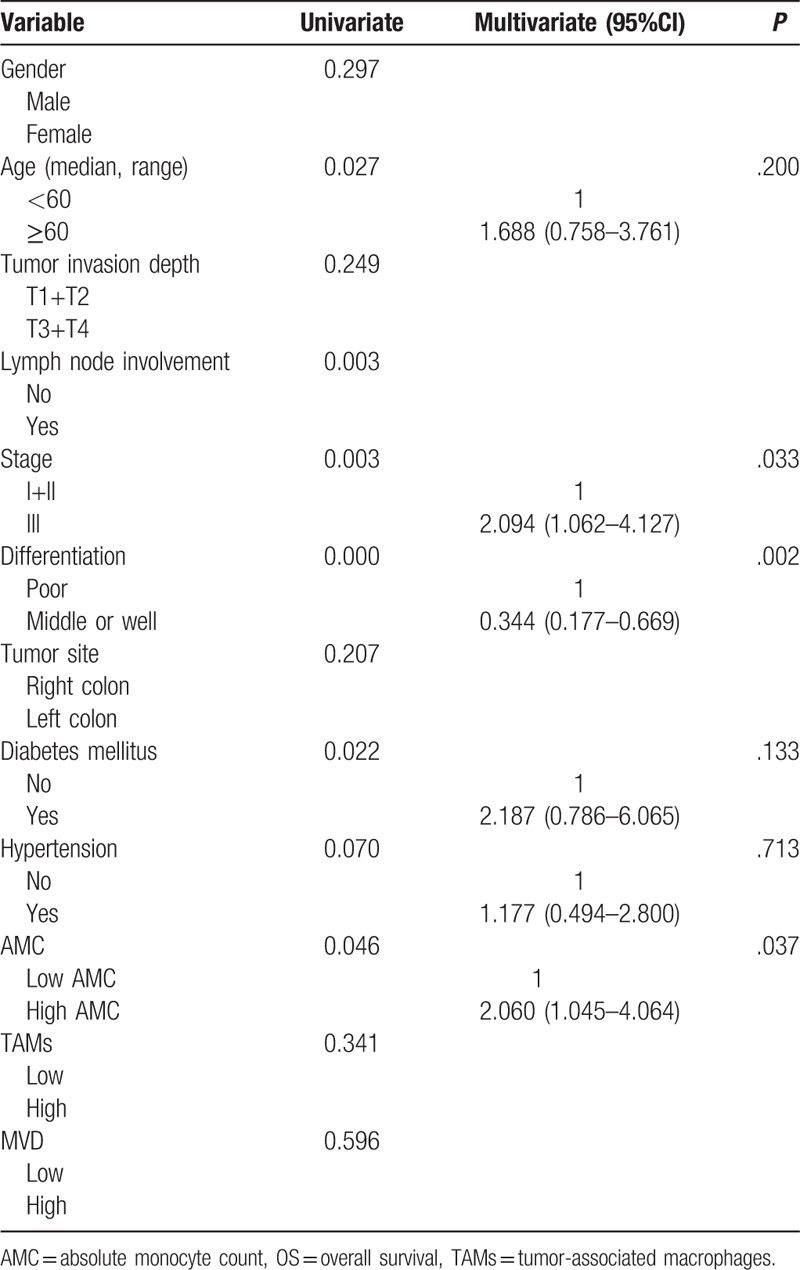
Univariate and multivariate analysis for OS.

**Table 3:**
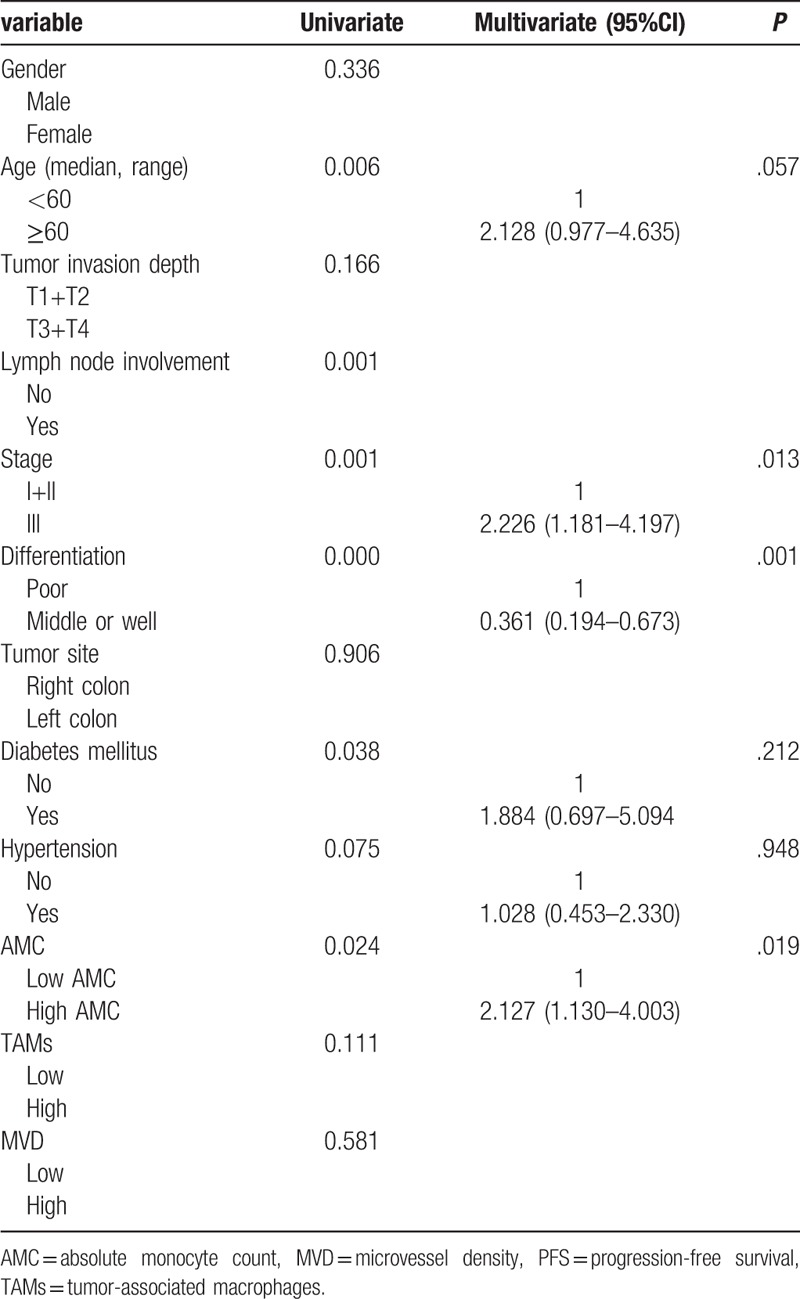
Univariate and multivariate analysis for PFS.

## 4 Discussion

The present study has shown that the preoperative AMC was an independent prognostic factor in colon caner; the high AMC was significantly associated with poor outcome. The result supported the hypothesis that monocyte plays an important role in cancer progression.

It was well known that monocytes can be divided into 2 main subsets: classical monocytes participating in inflammatory responses, nonclassical monocytes alleviating inflammatory properties and accelerating revascularization and wound healing.^[17]^ Several receptor–ligand pairs, such as CCL2/CCR2, CCL5/CCR5, CXCL12/ CXCR4, CX3CL1/CX3CR1, VEGFR1/VEGF-A, regulated the recruitment of monocytes into the tumor tissue.^[18]^ After migrating into tumor tissue, monocytes differentiated into tumor-associated macrophages. Classical monocytes induced by IFN-γ, LPS or TNF or GM-CSF generally differentiated into M1 macrophages playing an import role in proinflammation and tumor suppression. On the contrary, nonclassical monocytes induced by IL-4 and IL-13 differentiated into M2 macrophages playing immunosuppressive and tumor-promoting roles via TNF-alpha dependent MMP induction, production of interleukin (IL)-10,^[19]^ the programmed cell death-1 (PD-1)/programmed cell death-ligand 1 (PDL1) pathway.^[20,21]^

In previous studies, the peripheral monocyte count has been reported to be an useful prognostic factor in patients with various malignancies.^[7,14,22–25]^ Matsuo et al^[24]^ reported that peripheral AMC was significantly associated with myometrial tumor invasion, pelvic lymph node metastasis, clinical stage; elevated AMC was significantly associated with poor outcome in patients with endometrial cancer. Kumagai et al^[25]^ reported that peripheral AMC was significantly associated with poor outcome in lung adenocarcinoma, peripheral AMC significantly correlated with TAMs. Shibutani et al^[14]^ reported that the peripheral AMC was an useful prognostic factor in colorectal cancer. Hu et al^[7]^ reported that elevated peripheral AMC was significantly associated with poor outcome in colorectal cancer. Our study focused on colon cancer, furtherly testified the preoperative peripheral monocyte count was an independent prognosis in colon cancer.

The majority of previous studies showed that elevated TAMs was significantly associated with poor outcome in various malignancies, such as prostate cancer,^[26]^ lung cancer,^[27]^ hepatocellular carcinoma,^[28]^ and breast cancer.^[29]^ Based on the phenomenon that both peripheral monocyte count and TAMs were negatively associated with the outcome in various malignancies, it was postulated that the peripheral AMC was significantly associated with the density of TAMs in the tumor microenvironment. Until now, few studies^[14,25]^ have demonstrated that peripheral monocyte count significantly correlated with the density of TAMs. Nevertheless, there were ambivalent results in colorectal cancer. Some studies^[30]^ demonstrated that elevated TAMs was positively associated with better prognosis in colon cancer. Another studies demonstrated that elevated TAMs was significantly associated with poor prognosis in colorectal cancer. However, neither the correlation between TAMs and clinical outcome nor the direct correlation between AMC and TAMs were observed in our study.

The M2 TAMs had the ability of producing various proangiogenic factor including angiogenic chemokines VEGF, TGF-β, and PDGF, thereby promoting angiogenesis and cancer progression.^[31]^ There was general agreement that abnormal vasculatures play an important role in malignant aggressiveness, including tumor growth and metastasis.^[32]^ New vessel formation significantly increased the oxygen and nutrient for tumor cells. The vessels in tumors were divided into 2 types: pre-existing normal vessels and newly formed tumor vessel. CD34 has been considered as a marker of the tumor angiogenesis. Counting the endothelial vessels and measuring their density, also called MVD, was considered the most representative and useful tool for evaluating angiogenesis. As a matter of fact, the majority of previous studies supported the hypothesis that MVD plays a crucial role in cancer progression and prognosis. Nevertheless, several studies showed the opposite finding that MVD had no significant correlation with outcome and pathological features. The relationships between MVD and pathological features in colon cancer were still controversial. However, neither the correlation between MVD and pathological features nor direct correlation between TAMs and MVD were observed in our study.

The exact mechanism of the relationship between TAMs and angiogenesis was complex. Even though the majority of previous studies proved the direct correlation, our study was inconsistent with this theory. There were several explanations for this ambivalent phenomenon. First of all, the “hot spot” approach was the most common method for evaluating MVD and TAMs in various malignancies, areas of interest were selected at a low magnification (×100), and the density of TAMs and MVD was counted at high power field (×400 or ×200, respectively). The selective bias was not entirely eliminated. Second, there was no standardized method for quantification of TAMs and MVD levels. CD68 has been considered as a marker of the total tumor associated macrophages, but did not distinguish the M1 and M2 TAMs. CD34 was expressed not only in endothelial cells of small vessels, but also in inflammatory cells, lymphatic cells.

In conclusion, our results demonstrated that preoperative AMC was an independent prognosis in colon cancer, and elevated AMC was significantly associated with poor outcome. Based on the present evidences, neither the correlation between AMC and TAMs nor the correlation between TAMs and MVD were observed. It was emphasized that this conclusion should be confirmed in further study before making a final decision.

## Acknowledgments

All samples were collected from West China Hospital Biobank of Sichuan University. We thanked staffs (Department of West China Hospital Biobank) for offering assistance in the process of collecting samples. We also thanked staffs (Department of Pathology, The Second People's Hospital of Neijiang City, Sichuan Province) for help during the experiment.

## Author contributions

ZGL designed the study and wrote this manuscript. ZFX and ZGL conducted experiments. YQH and RZ collected the clinical data. RZ and YPC performed the statistical analysis. YZ and XTW reviewed the manuscript and proofread the final version. All the authors have read and approved the final manuscript.

**Conceptualization:** Rui Zhao.

**Investigation:** Zhaofen Xu, Yaping Cui.

**Resources:** Yuqian Huang.

**Supervision:** Yong Zhou, Xiaoting Wu.

**Writing – original draft:** Zhigui Li.
